# Active learning sequential surrogate-based optimization (AL-SSBO) versus response surface methodology in sunflower oil diacylglycerol synthesis: Performance and insight into AL-SSBO

**DOI:** 10.1016/j.fochx.2026.104516

**Published:** 2026-09-25

**Authors:** Haoran Liu, Yu Dai, Hong Liu, Siyao Zhang, Huanjun Yu, Qiqing Liao, Dong Chen, Xiaoming Jiang, Dongping He

**Affiliations:** a School of Food Science and Engineering, Wuhan Polytechnic University, Wuhan 430023, PR China; b Wuhan Institute for Food and Cosmetic Control, Wuhan 430040, PR China; c Baotou Jinlu Oil Co., Ltd., Baotou, Inner Mongolia 014000, PR China; d Wuhan Institute of Metrology, Standards and Quality, Wuhan 430050, PR China

**Keywords:** Diacylglycerol, Sequential surrogate-based optimization, Active learning, Process optimization

## Abstract

Diacylglycerol (DAG) are functional lipids with benefits including weight reduction and improved blood lipid profiles. However, enzymatic synthesis optimization is constrained by the rigid design of conventional response surface methodology (RSM). This study experimentally implemented and evaluated an Active-Learning Sequential Surrogate-Based Optimization workflow for sunflower oil DAG synthesis. AL-SSBO sequentially incorporated replicate measurements under recommended conditions and achieved a DAG content of 53.77% after five predefined optimization rounds. The AL-SSBO yielded a prediction error of only 0.10 percentage points, compared with 1.43 for RSM. Bootstrap-based inference supports the observed performance advantage of AL-SSBO over RSM. Optimal conditions were 11.93 h, 68.56 °C, 8.93 wt% lipase loading, and an oil-to-glycerol weight ratio of 11.71. Analysis of exploration–exploitation behavior and spatial sampling showed that the recommended conditions became increasingly concentrated near a high-DAG region, consistent with retrospective stabilization within the investigated domain.

## Highlights

• Outperforms RSM in experimental response and prediction accuracy.
• Distance-based exploration and K-means diversification improve sample efficiency.
• A five-round closed loop enables experimental validation and adaptive model updating.
• Optimization trajectories reveal how sampling decisions guide process exploration.
• XGBoost achieves the highest cumulative best experimental DAG content.

## Graphical abstract

**Figure:**
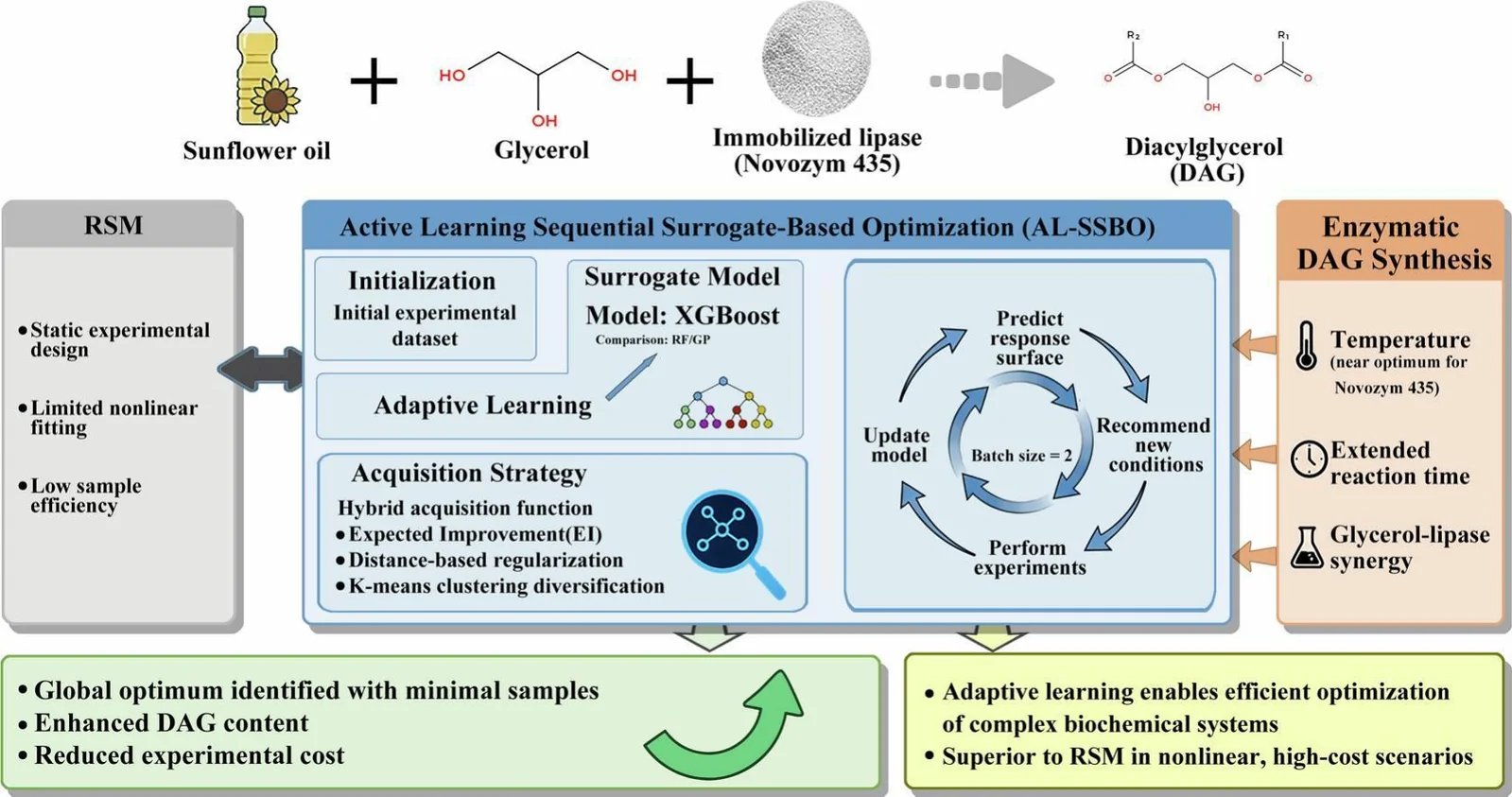

## 1 Introduction

Diacylglycerol (DAG) exerts beneficial effects on metabolic health by modulating lipid metabolic pathways, highlighting its significant potential for preventing obesity and associated metabolic disorders (Jollet et al., 2024; Lee et al., 2020; Yanai et al., 2007). As a minor natural component of vegetable oils, DAG is generally recognized as safe (GRAS) for human consumption. However, its natural abundance typically accounts for less than 10% of the oil (Feng et al., 2022), necessitating exogenous synthesis to achieve functionally effective concentrations. Numerous studies have reported on the production of DAG using various triglyceride feedstocks, such as olive oil, animal fats, and other lipid sources, through chemical or enzymatic approaches (Feltes et al., 2012; Hu et al., 2023; Wang et al., 2011; Yin et al., 2025; Zhou et al., 2025). Enzymatic glycerolysis, which involves the lipase-catalyzed reaction of triglycerides with glycerol at elevated temperatures, has proven particularly effective in producing high-purity DAG with enhanced yield (Diao et al., 2017; Lo et al., 2007; Zheng et al., 2023). Sunflower oil exhibits excellent bioavailability, with a digestion and absorption rate exceeding 96% in humans, and is rich in bioactive components, including tocopherols, phytosterols, and essential trace elements (Nakonechna et al., 2024; Petraru et al., 2021; Vicentini-Polette et al., 2024; Xu et al., 2022), rendering it a highly suitable and nutritionally favorable feedstock for DAG synthesis.

In the field of food engineering, feature-based process optimization plays a pivotal role in improving product performance, yield, and economic efficiency. The enzymatic synthesis of DAG involves four interdependent features: lipase loading, substrate weight ratio, temperature and time (Guo et al., 2025). This process is conventionally optimized using response surface methodology (RSM). This approach begins by defining a parameter space based on prior knowledge, followed by the simultaneous execution of all experimental runs using structured designs such as central composite design (CCD) or Box-Behnken design. The experimental data are then used to fit an explicit second-order polynomial model, establishing a functional relationship between the input parameters and response variables. The optimal parameter combination is then identified through mathematical optimization techniques applied to the fitted model. Owing to its well-defined framework and procedural standardization, RSM has been extensively employed in various engineering and scientific domains over the past several decades (Guebara et al., 2018).

As the complexity of optimized systems continues to increase, the theoretical and practical limitations of RSM in addressing modern high-dimensional and nonlinear process optimization have become increasingly pronounced. At the model representation level, RSM is a parametric model widely used in biology and chemistry (Huang et al., 2021; Laougé et al., 2020; Mohammed et al., 2020). However, this modeling approach inherently struggles to characterize the high-order nonlinearities, complex interaction effects, and non-monotonic response relationships commonly encountered in real chemical processes. The overall approximation quality can be assessed using statistical indicators, but difficulties in establishing appropriate model constraints may lead to discrepancies between predicted and experimental values (Gavin & Yau, 2008). In these circumstances, it is difficult to guarantee the reliability of gradient optimization or extremum inference based on the model. Therefore, the final optimization results are subject to significant uncertainty. At the experimental strategy level, RSM follows a static approach in which the experimental design is determined before model building. The spatial distribution of experimental points is entirely determined by the initial design and cannot be dynamically adjusted based on new information acquired during the experiment (Zhao et al., 2017). This strategy results in low sample efficiency, as a substantial portion of experimental resources is consumed in confirming the range. Potential optimal regions may be overlooked if they are not included in the initial design space, thereby significantly increasing the risk of optimization failure and escalating experimental costs (Pruksawan et al., 2019; Raponi et al., 2021). RSM may introduce a data compression step during modeling when replicate experiments are averaged, which removes the deviations among them and filters out the second-moment information (variance) of the underlying distribution before it enters the main model. In essence, replicate observations are thereby reduced to point estimates of the conditional mean. This dimensionality reduction imposes a strong conditional-mean assumption—the variability among parallel samples is used only for a single noise estimate and does not inform model structure inference, thereby discarding potentially valuable information about the response surface's local uncertainty. These limitations of RSM—far from isolated—compound one another, constraining its effectiveness and reliability in modern complex system optimization and highlighting the urgent need for more adaptive, learning-capable intelligent optimization methods (Lim et al., 2025; Mousa et al., 2022).

Machine learning models have been applied to lipid-related conversion, synthesis, and product characterization. Gaussian process (GP) regression has been used to predict biodiesel yields during the transesterification of mustard oil and papaya oil under different reaction conditions (Kangani et al., 2024; Sumayli & Alshahrani, 2023). GP-based approaches depend on the selected covariance function, and repeated model fitting can increase computational demand during sequential optimization. Random forest (RF) has been evaluated for predicting the sliding melting point of structured lipids prepared by enzymatic interesterification of coconut oil and palm stearin (Li et al., 2026). RF can represent nonlinear relationships and interactions among process variables, although its tree-based predictions may generate less smooth response surfaces. In addition, RF and extreme gradient boosting (XGBoost) have been used to predict glycerol monolaurate production in a related glycerol-ester synthesis system (Mehta et al., 2026). XGBoost can capture nonlinear factor–response relationships and incorporates regularization, although its performance depends on hyperparameter settings and data availability. However, although GP, RF, and XGBoost can serve as surrogate models, their predictive outputs alone do not define how subsequent experimental conditions should be selected and experimentally evaluated. Moreover, the use of these models within experimentally guided sequential optimization for enzymatic diacylglycerol production remains limited.

Sequential Surrogate-Based Optimization has emerged as a powerful and versatile approach to address diverse design challenges. It is widely employed in robotics (Martinez-Cantin et al., 2007; Wang et al., 2016), machine learning (Hu et al., 2025), experimental design (Branco-Katcher et al., 2026; Siska et al., 2026), and optimization tasks (Jin & Xu, 2025; Tian et al., 2025). In recent years, researchers have increasingly applied machine learning to overcome the limitations of RSM in food process optimization (Babatunde et al., 2025; Mukherjee et al., 2025). By dynamically constructing probabilistic surrogate models and integrating sequential experimental design, this method enables accurate predictions in data-rich regions of the parameter space while quantifying uncertainty in sparsely sampled areas (Sekulic et al., 2025). This dual capability effectively addresses the two main shortcomings of RSM in complex nonlinear systems: model underfitting and inefficient exploration. This enhances the system's predictive accuracy and optimization efficiency. In this study, the integrated data-driven Active Learning Sequential Surrogate-Based Optimization (AL-SSBO) (Guan et al., 2024; Kohl et al., 2024) is examined, forming a synergistic framework that demonstrates significant application potential. Within the AL-SSBO, each experiment transcends isolated trial-and-error exploration. This mechanism ensures that valuable experimental resources are dynamically and adaptively allocated to regions that either offer the greatest potential for performance improvement or improve the coverage of less-sampled regions, thereby achieving substantially higher sample efficiency. Unlike the static and retrospective modeling paradigm of RSM, the proposed approach establishes a dynamic, sequential iterative optimization loop comprising “prediction – recommendation – experiment – update” (Li et al., 2025; Park et al., 2024; Shields et al., 2021), enabling adaptive decision-making through continuous model refinement based on feedback. The AL-SSBO strategy makes full use of sample information.

In sunflower oil DAG production, the CCD dataset served as a common starting point for the RSM model and the initial AL-SSBO model. The contribution of this study lies not in proposing a new surrogate-learning algorithm, but in experimentally implementing and evaluating a five-round prediction–recommendation–experiment–update workflow for enzymatic DAG synthesis. The sequential procedure integrated surrogate prediction, distance-based exploration, batch diversification, and experimental feedback, enabling the sampling decisions and optimization performance to be examined at the process level within the investigated factor domain.

## 2 Material and methods

### 2.1 Material

Sunflower seed oil was purchased from Yihai Kerry Arawana Holdings Co., Ltd. Glycerol was obtained from Sinopharm Chemical Reagent Co., Ltd. (Shanghai, China). Immobilized lipase Novozym 435 (*Candida antarctica* lipase B immobilized on macroporous acrylic resin; esterification activity: 10,000 U/g) was provided by Novozymes (China) Biotechnology Co., Ltd. High-performance liquid chromatography (HPLC)-grade n-hexane, isopropanol, and formic acid were acquired from Tianjin Kemiou Chemical Reagent Co., Ltd. All other solvents and reagents were of analytical grade unless otherwise specified.

A SHA-B digital constant temperature water bath shaker (Saidelisi Experimental Analytical Instrument Manufacturing Plant, China), a LT200B electronic analytical balance (Changshu Tianliang Instrument Co., Ltd., China), a VXMNFS vortex mixer (Ohaus Instruments (Shanghai) Co., Ltd., China), a DH18B centrifuge (Hunan Duoheng Instrument Equipment Co., Ltd., China), and a Waters 2695 high-performance liquid chromatography (HPLC) system equipped with a Waters 2414 refractive index detector (Waters Corporation, USA) and a silica column (4.6 mm × 250 mm, 5 μm; Phenomenex, USA) were used in this study.

### 2.2 Methods

#### 2.2.1 Preparation of sunflower seed oil diacylglycerol

The synthesis of DAG was conducted based on the dataset containing various process parameters to predict the DAG yield. 10 g of sunflower seed oil was placed in a 50 mL container and sterilized at 85 °C for 10 min. A specific amount of glycerol was then added, and the mixture was homogenized thoroughly using a high-speed homogenizer. Subsequently, a predetermined amount of Novozym 435 was introduced, and the reaction was carried out in constant temperature water bath shaker for a specified duration. For sample analysis, aliquots were withdrawn and centrifuged at 5000 rpm for 15 min. The upper oil phase was collected, yielding the final sunflower seed DAG product.

#### 2.2.2 Analytical methods

The sunflower oil DAG content was determined using High-Performance Liquid Chromatography (HPLC) equipped with a Waters 2414 RID and Silica gel column (4.6 mm × 250 mm × 5 μm, Phenomenex, USA) maintained at 30 °C. The mobile phase consisted of n-hexane, isopropanol, and formic acid in a volume ratio of 15:1:0.003 (v/v/v), which was delivered at a constant flow rate of 1.0 mL/min. The detector temperature was set at 35 °C, while the autosampler tray was maintained at 25 °C. Use the above mobile phase as the solvent to prepare a sunflower seed oil DAG sample at a concentration of 2 mg/mL, then vortex mix. Each sample (30 μL) was filtered through a 0.22 μm nylon-based filter membrane, with an injection runtime of 30 min. The DAG concentration was quantified using the peak area normalization method.

(1) $$W_{\mathrm{DAG}}\left(\%\right)=\frac{A_{1,3-\mathrm{DAG}}+A_{1,2-\mathrm{DAG}}}{A_{1,3-\mathrm{DAG}}+A_{1,2-\mathrm{DAG}}+A_{\mathrm{TAG}}+A_{\mathrm{MAG}}+A_{\mathrm{FFA}}}\times 100\;$$

#### 2.2.3 Central composite design

Compared with a three-level full factorial design requiring 3^4^ = 81 runs, the 30-run rotatable CCD provided the information needed to estimate linear, two-factor interaction, and quadratic effects, together with pure experimental error from the replicated center points, while maintaining a practicable experimental burden. Preliminary single-factor experiments were conducted to examine the effects of reaction time, temperature, lipase loading, and substrate weight ratio and to guide the selection of the factor ranges used in the CCD. A rotatable CCD was employed to characterize the effects of the four process variables on DAG content and to support fitting of the second-order response surface model. The experimental design comprised 16 factorial points, 8 axial points (α = 2.0), and 6 center points, resulting in a total of 30 experimental runs. Each factorial and axial point was evaluated in one independent reaction, whereas the center condition was independently repeated six times. The factorial and axial points enabled the estimation of linear, interaction, and quadratic terms, whereas the replicated center points provided an estimate of pure experimental error. The resulting CCD dataset was used for RSM modeling and subsequently provided the experimental data for the AL-SSBO procedure. The independent variables and their levels for the CCD are summarized in Table 1.

**Table 1:** The independent variables and levels for CCD.

Level	Factors			
	$X_{1}$ Time/h	$X_{2}$ Temperature/°C	$X_{3}$ Lipase loading/%	$X_{4}$ Substrate weight ratio
−2	9	50	6	9
−1	10	60	7	10
0	11	70	8	11
1	12	80	9	12
2	13	90	10	13

At each axial point (±α), only one factor was set at its axial level, while the other three factors were maintained at their center levels. The specific combinations used in the CCD are presented in Table S1.

In this regression model, four input variables were selected as predictive factors: time ($X_{1}$, h), temperature ($X_{2}$, °C), lipase loading ($X_{3}$, wt%, *w*/w), and the Substrate weight ratio ($X_{4}$). Substrate weight ratio is defined as the ratio of the weight of oil to the weight of glycerol($X_{4}=\frac{w_{\text{oil}}}{w_{\text{glycerol}}}$), was expressed as a single numerical value.

#### 2.2.4 Second-order polynomial model for RSM

In RSM, the optimization objective was the content of DAG (Y, %). A second-order polynomial model was fitted using the least squares method:

(2) $$Y=\beta_{0}+\sum \beta_{i}X_{i}+\sum \beta_{i}X_{i}^{2}+\sum \beta_{\mathrm{ij}}X_{i}X_{j}+\varepsilon \;$$

i, j = 1,2,3,4….

#### 2.2.5 Theory of active learning sequential surrogate-based optimization

This study employed a dynamic and adaptive AL-SSBO framework as the machine learning approach, which was designed to explore the process parameter space and identify favorable conditions within the investigated experimental domain through an iterative, closed-loop workflow (Branco-Katcher et al., 2026). The overall procedure was structured as a sequential decision-making process comprising 5 rounds and a total of 10 recommended experimental conditions.

From a total of 30 experimental data points, 80% were randomly selected to form the initial training set, while the remaining 20% were reserved as a test set. Before generating the first-round AL-SSBO recommendations, the selected surrogate model was fitted using the initial training set. Each iteration constituted a complete cycle, repeated over 5 rounds. In each round, the accumulated experimental data were randomly divided at an 80:20 ratio, and an XGBoost regression model was trained on the resulting training subset and served as the core surrogate model for guiding subsequent recommendations.

XGBoost is an efficient and scalable machine learning algorithm built upon the gradient boosting framework. It improves model performance and computational efficiency by systematically constructing ensembles of decision trees and incorporating second-order gradient information through Taylor expansion, along with structured regularization terms. Due to its robustness and high predictive accuracy, XGBoost has become widely adopted in regression tasks (Sánchez et al., 2025). Since its introduction in 2016, it has been successfully applied across diverse scientific domains, including chemistry (Li et al., 2022), materials science (Dean et al., 2023), biology (Alam et al., 2025), medicine (Zhang et al., 2023), and food science (Chen et al., 2025).

XGBoost's fundamental innovation is the explicit integration of tree complexity into the objective function, which allows for cooperative optimization of the loss function and regularization components. This approach can reduce overfitting and increases model generalization.

XGBoost trains an ensemble of K regression trees in an additive and iterative fashion. The predicted output at the **t-**th iteration is expressed as:

(3) $${\hat{y}}_{i}^{\left(t\right)}={\hat{y}}_{i}^{\left(t-1\right)}+f_{t}\left(x_{i}\right)$$

where $f_{t}$ is the newly learned tree at iteration t. The objective function consists of a loss term and a regularization term:

(4) $$L^{\left(t\right)}=\sum_{i=1}^{n}l\left(y_{i},{\hat{y}}_{i}^{\left(t-1\right)}+f_{t}\left(x_{i}\right)\right)+\Omega \left(f_{t}\right)\;$$

with

(5) $$\Omega \left(f_{t}\right)=\mathrm{\gamma T}+\frac{1}{2}\lambda \sum_{j=1}^{T}w_{j}^{2}\;$$

where T denotes the number of leaf nodes, $w_{j}$ is the output value (weight) of the j-th leaf, and γ and λ are regularization coefficients controlling the tree structure and leaf weights, respectively. This allows for the analytical derivation of the optimal leaf weights and structure score, which greatly accelerates training and improves model accuracy.

The main advantages of XGBoost are its exceptional computational efficiency and its ability to handle missing values. It also has a flexible parameter space that supports randomization strategies such as column and row subsampling, which help to mitigate overfitting. However, the algorithm has numerous hyperparameters (e.g., tree depth, learning rate, and regularization coefficients), which makes the tuning process relatively complex and sensitive to dataset size.

A 5 × 5 nested cross-validation approach was employed to perform internal validation. The dataset was iteratively partitioned into distinct training and validation subsets. The outer cross-validation loop was utilized to assess the model's average predictive performance ($\mu_{\mathrm{CV}}$), whereas the inner loop facilitated hyperparameter tuning through simulation.

The system then advances to the AL-SSBO iteration phase, building upon the trained surrogate model. The primary objective of this phase is to identify and recommend the next batch of experimental points that balance predicted performance and spatial exploration. The acquisition function used in this study integrates a prediction-based exploitation term and a distance-based exploration reward with a distance-based regularization mechanism and a K-means clustering-based diversification strategy. The scoring criterion establishes a dynamic balance between exploiting regions with high predicted performance and exploring regions far from existing training samples, by combining the surrogate model's predicted value with a distance-based exploration bonus (scaled by the average nearest-neighbor distance in the training set) and a large penalty for candidates exceeding a predefined distance threshold. After evaluating hundreds of randomly sampled candidates using this composite scoring function, the algorithm partitions the most promising candidates into K clusters (In this study, K = 2.) based on their spatial distribution in the parameter space. The final recommendations are the highest-scoring point in each cluster, ensuring high predicted performance and spatial diversity.

The distance constraint acts as an additional penalization term, discouraging the selection of candidates that exceed the predefined distance threshold. This triple mechanism—combining prediction-based exploitation, K-means diversification, and distance penalization—improves the diversity and safety of the proposed experimental conditions, reducing the allocation of resources to unnecessary or high-risk trials. Because of these mechanisms, the model rapidly leaps between widely separated regions of the feature space from one iteration to the next, making exploration far more efficient. By maximizing this enhanced acquisition function through sequential optimization algorithms, the system automatically selects and outputs two candidate experimental conditions ($x_{k,1}$, $x_{k,2}$) in each round, which balance between performance potential and exploratory diversity.

The optimization proceeded in 5 rounds (iterations), with 2 experimental conditions recommended per round (batch size = 2). The two recommended experimental conditions were experimentally validated. Each recommended condition was evaluated in three independent reaction replicates. The corresponding measured DAG content values ($y_{k,1}$, $y_{k,2}$) were all incorporated into the cumulative training dataset, thereby completing one full iteration of the data feedback loop. Each round allowed the surrogate model to assimilate the newly acquired experimental data for use in the next round. No formal blocking or experimental run-order randomization was applied. The five AL-SSBO rounds were conducted sequentially because the recommendations in each round depended on the experimental results obtained in the preceding rounds.

To evaluate surrogate-model selection, the AL-SSBO procedure was additionally implemented using Gaussian process (GP) and random forest (RF) models. All three procedures started from the same initial CCD dataset and used the same factor ranges, candidate-selection strategy, number of optimization rounds, number of recommended conditions per round, and number of experimental replicates. Each model was updated only with the experimental results obtained under its own recommended conditions. Prediction errors were assessed along each model-specific optimization trajectory and, for direct comparison, on a common condition-level dataset using the same repeated cross-validation partitions.

#### 2.2.6 Retrospective assessment of AL-SSBO stabilization

Five AL-SSBO rounds were defined in advance as the experimental budget and were not terminated according to a prespecified adaptive stopping rule. After all five rounds had been completed, a retrospective assessment was conducted to determine whether the results obtained by the final round supported stabilization of the sequential optimization. For the initial CCD dataset and each cumulative dataset obtained after Rounds 1–5, 10,000 condition-level bootstrap samples were generated by resampling experimental conditions with replacement while retaining the replicate measurements from each condition within the same resampled unit. For each bootstrap sample, the XGBoost model was refitted using the parameter settings selected for the corresponding cumulative round, and the model-predicted optimum was identified from a fixed candidate set within the originally defined search domain. The bootstrap distribution of the model-predicted optimum was summarized by its mean, 95% bootstrap percentile interval, relative interval width, and coefficient of variation. Stabilization was assessed retrospectively using three descriptive checks. First, the gain in cumulative best experimental DAG content did not exceed the pooled standard error of a triplicate condition mean. Second, the absolute one-step prediction error did not exceed the pooled within-condition standard deviation. This error was calculated from the prediction recorded before the final round and the experimental mean of its best-performing condition. Third, the best-performing condition in the final round was identical to that in the preceding round. The relative interval width and coefficient of variation were additionally calculated to describe the dispersion of the bootstrap-predicted optimum.

## 3 Results and discussion

### 3.1 Central composite design and response surface experimental optimization

As shown in Fig. S1, the preliminary single-factor experiments indicated comparatively high DAG contents at approximately 11 h, 70 °C, 8 wt% lipase loading, and an oil-to-glycerol weight ratio of 11. These values were therefore adopted as the corresponding center levels in the CCD. The step sizes corresponding to one coded unit were set at 1 h, 10 °C, 1 wt% lipase loading, and one unit of weight ratio. With an axial distance of α = 2.0, the axial points extended the CCD domain to 9–13 h, 50–90 °C, 6–10 wt% lipase loading, and oil-to-glycerol weight ratios of 9–13. The complete CCD design is presented in Table 1. The fitted models and subsequent optimization results were interpreted only within this experimentally investigated domain.

The experimental design and results of the CCD are shown in Table S1. The ANOVA results for the regression model are presented in Table 2. The combined 3D response surfaces and 2D contour plots are illustrated in Fig. 1.

**Table 2:** ANOVA for the regression model.

Source of variation	Sum of squares	df	Mean square	F-value	*P*-value	Significance
Model	273.87	14	19.56	26.50	< 0.000 1	**
$X_{1}$ (time/h)	11.77	1	11.77	15.95	0.001 2	**
$X_{2}$ (temperature/°C)	27.12	1	27.12	36.73	< 0.000 1	**
$X_{3}$ (lipase loading/%)	26.27	1	26.27	35.58	<0.000 1	**
$X_{4}$ (substrate weight ratio)	2.57	1	2.57	3.48	0.081 9	
$X_{1}X_{2}$	13.86	1	13.86	18.77	0.000 6	**
$X_{1}X_{3}$	5.65	1	5.65	7.66	0.014 4	*
$X_{1}X_{4}$	3.87	1	3.87	5.24	0.036 9	*
$X_{2}X_{3}$	0.011	1	0.011	0.014	0.970 3	
$X_{2}X_{4}$	0.20	1	0.20	0.27	0.614 1	
$X_{3}X_{4}$	1.48	1	1.48	2.01	0.176 9	
$X_{1}^{2}$	19.47	1	19.47	26.38	0.000 1	**
$X_{2}^{2}$	68.16	1	68.16	92.31	<0.000 1	**
$X_{3}^{2}$	17.07	1	17.07	23.12	0.000 2	**
$X_{4}^{2}$	130.07	1	130.07	176.17	< 0.000 1	**
Residual	11.07	15	0.74			
Lack of fit	8.87	10	0.89	2.01	0.227 9	
Pure error	2.20	5	0.44			
Cor total	284.95	29				

**Note:** ** represents extremely significant (*P* < 0.01); * represents significant (*P* < 0.05). Coefficient of determination $R^{2}$ = 0.9611; $R_{\mathrm{adj}}^{2}$ = 0.9249.

**Fig. 1:**
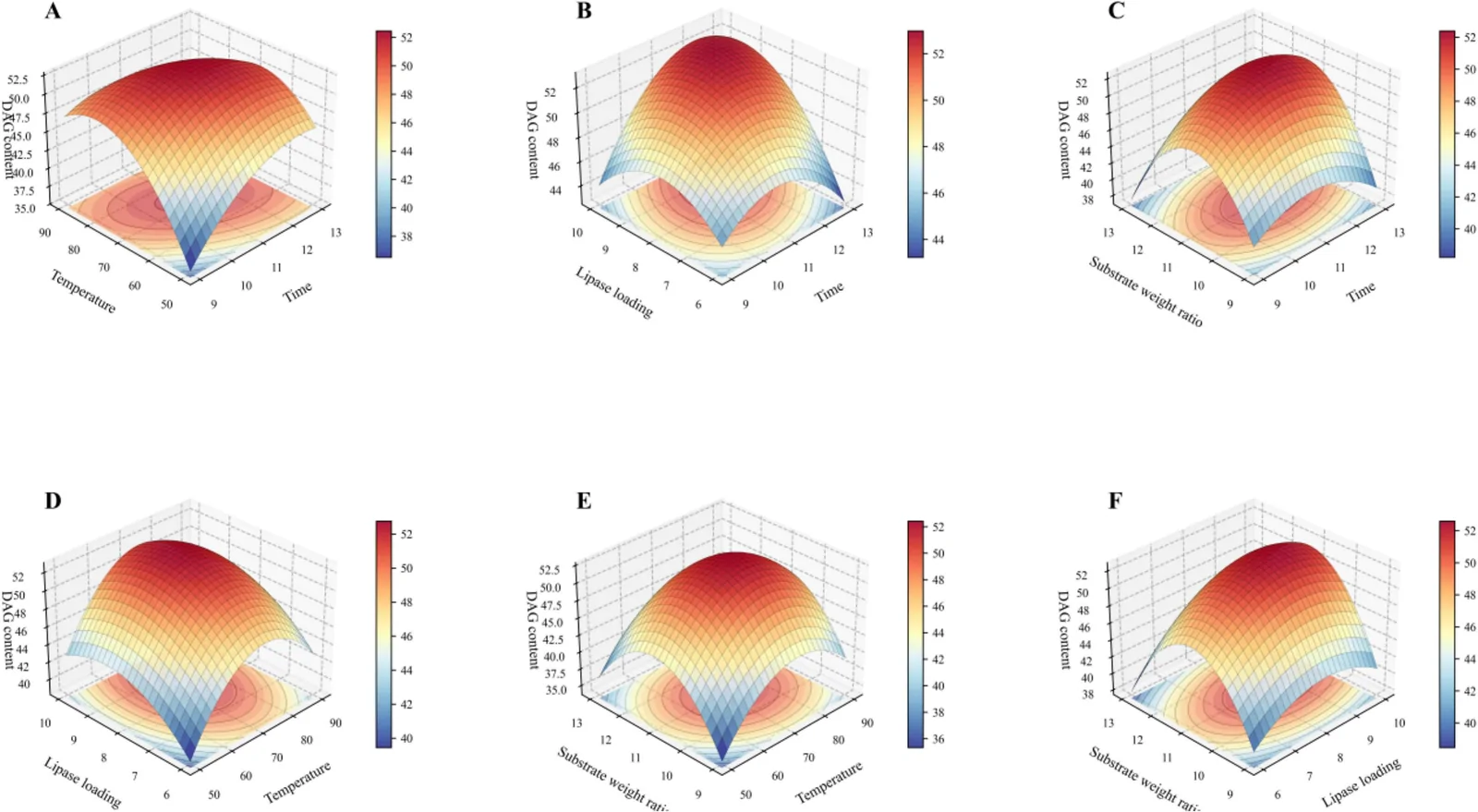
The combined 3D response surfaces and 2D contour plots: (A) Temperature and time vs. DAG content. (B) Lipase loading and time vs. DAG content. (C) Substrate weight ratio and time vs. DAG content. (D) Lipase loading and temperature vs. DAG content. (E) Substrate weight ratio and temperature vs. DAG content. (F) Substrate weight ratio and lipase loading vs. DAG content.

Multiple linear regression analysis was performed using Design Expert software to obtain the regression model equation:

(6) $$\begin{array}{c} Y=52.24+0.7X_{1}+1.06X_{2}+1.05X_{3}+0.33X_{4}-0.93X_{1}X_{2}+0.59X_{1}X_{3}+0.49X_{1}X_{4}\\ -0.08X_{2}X_{3}-0.11X_{2}X_{4}+0.3X_{3}X_{4}-0.84X_{1}^{2}-1.58X_{2}^{2}-0.79X_{3}^{2}-2.18X_{4}^{2} \end{array}$$

The model significance was evaluated by analysis of variance (ANOVA).

### 3.2 Active learning sequential surrogate-based optimization process

The optimization results for XGBoost-based AL-SSBO are shown in Table 3.

**Table 3:** Optimization results for XGBoost-based AL-SSBO.

Iterative round	Time (h)	Temperature (°C)	Lipase loading (wt%)	Substrate weight ratio	DAG content (%)	
					Predicted	Actual
Round 1	10.26	69.01	8.91	11.21	52.79	49.56
	10.50	63.13	8.10	11.43	52.71	51.27

Round 2	11.08	77.85	8.61	11.79	51.35	45.67
	11.70	70.40	8.10	11.12	51.78	51.34

Round 3	11.00	70.23	8.60	11.30	51.42	52.76
	11.43	62.39	8.52	11.12	50.87	51.58

Round 4	11.59	62.79	8.24	11.07	51.34	51.42
	11.93	68.56	8.93	11.71	53.03	53.87

Round 5	11.04	62.80	8.75	11.48	51.83	51.60
	11.93	68.56	8.93	11.71	53.87	53.77

Once all the optimization iterations had been completed, the machine learning workflow proceeded to the parameter refinement stage. A final XGBoost model was trained using the experimental data (30 CCD samples +30 AL-SSBO samples = 60 samples) accumulated throughout the process.

### 3.3 AL-SSBO performance analysis

To evaluate the performance of AL-SSBO, multiple assessment metrics were analyzed. These include iterative prediction effectiveness, stability analysis, and sample efficiency evaluation, among others.

Predicted and actual DAG contents for both conditions recommended in each AL-SSBO round are shown in Fig. 2. The two conditions within each round are displayed as a group because they were recommended at the same stage and were jointly incorporated into the subsequent model update, rather than representing consecutive experimental points. The MAE for the two conditions were 2.33, 3.06, 1.03, 0.46, and 0.16 percentage points in Rounds 1–5, respectively. Although the prediction errors did not decrease monotonically during the early rounds, the agreement between predicted and experimental values improved from Round 2 onward. Although C7 and C9 yielded lower experimental DAG contents than C5, C5, C7, and C9 do not form a sequential trajectory. Instead, the simultaneous evaluation of the lower-response conditions C7 and C9 and the higher-response conditions C8 and C10 is consistent with the exploration–exploitation design of AL-SSBO, in which spatially diverse conditions were evaluated alongside candidates with high predicted responses. The absolute prediction errors were 0.08, 0.84, 0.23, and 0.10 percentage points for C7, C8, C9, and C10, respectively. Thus, the lower experimental DAG contents of C7 and C9 do not indicate deterioration of the model's predictive performance in the later rounds.

**Fig. 2:**
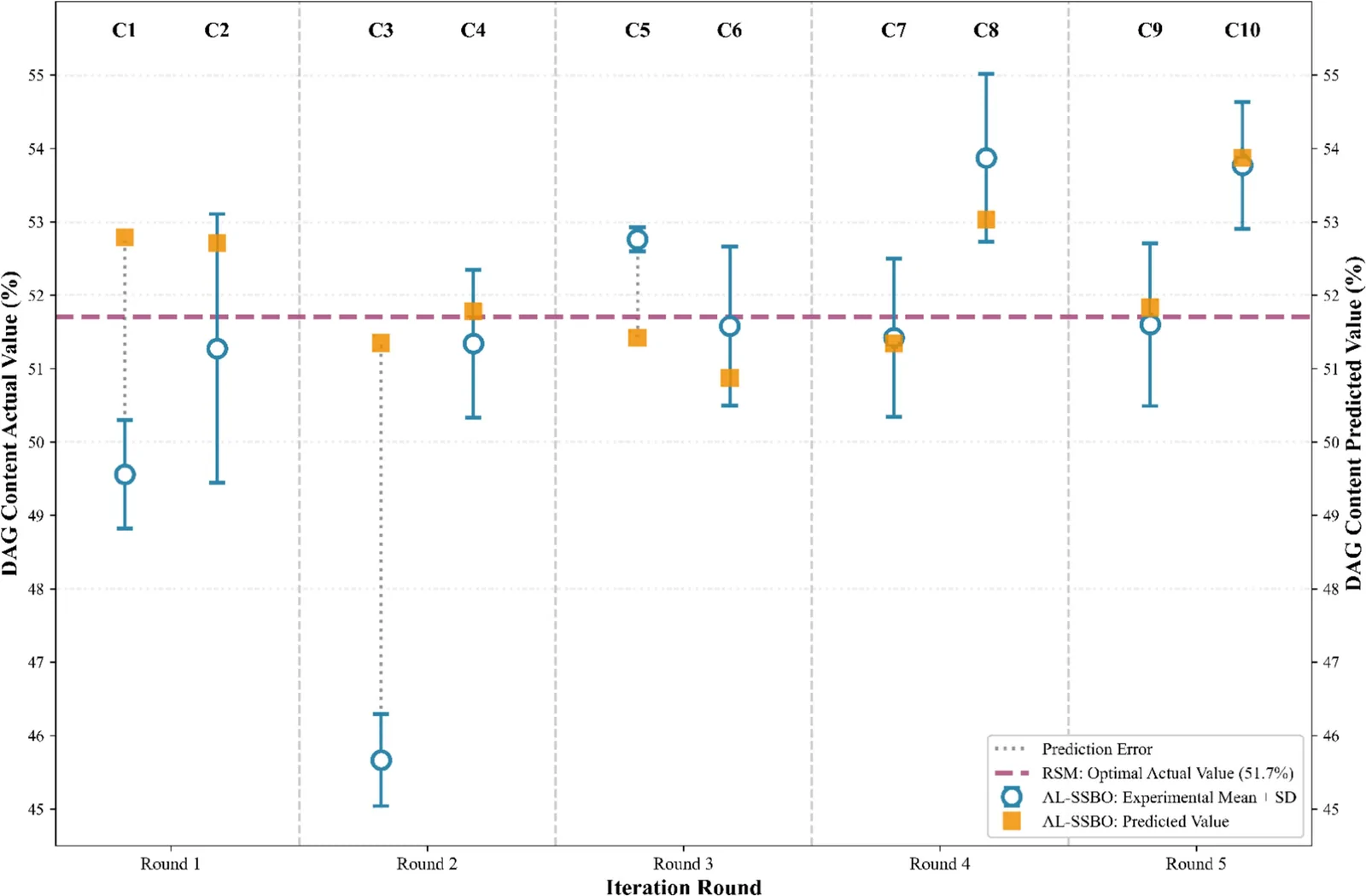
Predicted and experimental DAG contents for the ten AL-SSBO recommendations, with the experimental RSM reference. The two conditions recommended in each round are displayed side by side, without implying an order within the round. Experimental values are presented as means ± SD (*n* = 3). Dashed vertical lines separate successive rounds. Dotted lines connect each prediction with its corresponding experimental mean to indicate the prediction error. The dashed horizontal line indicates the experimental RSM reference of 51.70 wt%.

The experimental results summarized in Table 4 demonstrate the repeatability of the XGBoost-based AL-SSBO procedure. The experimental system demonstrated excellent repeatability, with a pooled standard deviation of 1.046, a corresponding standard error of 0.604, and a mean relative standard deviation of 1.88%. This indicates high precision and robust control over the analytical procedures. Relative standard deviation below 5% indicate acceptable method precision.

**Table 4:** Data comparison of DAG content during the AL-SSBO process.

Iterative round	Condition	Rep 1	Rep 2	Rep 3	Mean	Std	RSD (%)
Round 1	C1	49.66	48.78	50.24	49.56	0.735	1.483
	C2	49.20	52.67	51.95	51.27	1.831	3.572

Round 2	C3	46.37	45.17	45.46	45.67	0.626	1.371
	C4	51.59	50.23	52.20	51.34	1.009	1.964

Round 3	C5	52.66	52.67	52.95	52.76	0.165	0.312
	C6	51.58	50.50	52.66	51.58	1.080	2.094

Round 4	C7	51.51	50.30	52.45	51.42	1.078	2.096
	C8	52.55	54.50	54.56	53.87	1.144	2.123

Round 5	C9	52.72	50.50	51.58	51.60	1.110	2.151
	C10	53.62	52.99	54.70	53.77	0.865	1.608

Note: Mean, Std, and Rep values are given in % (mass fraction); RSD values are in %.

The GP- and RF-based sequential optimization results and their comparison with XGBoost are presented in Tables S2 and S3 and Fig. S2. On the shared condition-level dataset, RF achieved the lowest cross-validation MAE (1.62 ± 0.44 percentage points), whereas RF and GP showed the same lowest cross-validation RMSE (2.24 ± 0.61 percentage points). The corresponding MAE values were 1.64 ± 0.38 percentage points for GP and 1.62 ± 0.44 percentage points for RF, while the XGBoost was 2.02 ± 0.46 percentage points. Across the separately realized model-specific five-round sequential trajectories, GP achieved the lowest overall sequential MAE and RMSE (0.93 and 1.24 percentage points, respectively), followed by RF (1.26 and 1.53 percentage points) and XGBoost (1.41 and 2.19 percentage points). However, XGBoost achieved the lowest final-round sequential MAE and RMSE (0.16 and 0.18 percentage points) and the highest cumulative best experimental DAG content (53.87 wt%), compared with 53.12 wt% for GP and 52.84 wt% for RF. XGBoost was therefore retained as the surrogate model for the sequential optimization objective in the present system based on its final-round performance and experimentally achieved response, without claiming universal superiority in predictive performance.

To validate that the observed performance variations were driven by systematic optimization rather than experimental artifacts, we examined the signal-to-noise ratio. The value of 7.84 confirms that the condition-dependent variations reflect genuine optimization signals. The AL-SSBO single-factor ANOVA yielded an F-statistic of 15.16 (*p* < 0.0001), demonstrating that the variance between conditions was 15 times that of the experimental error within conditions. Such a high variance ratio provides compelling evidence that AL-SSBO successfully identified optimal parameter combinations.

The optimal condition (C8) produced a mean DAG content of 53.87%, with replicate values ranging from 52.55% to 54.56%. Using the t-distribution with two degrees of freedom, the 95% confidence interval was [51.03%, 56.71%]. The mean within-condition relative standard deviation for C7–C10 was 1.995%, indicating consistent repeatability during the final two optimization rounds. This exceptional consistency indicates that minor parameter fluctuations do not result in catastrophic performance degradation.

To quantitatively evaluate the sample efficiency of the AL-SSBO framework, its performance was compared against a random sampling benchmark. The random sampling benchmark simulated 1000 independent random experiments using an XGBoost model trained on actual experimental data. Each experiment comprised 10 randomly generated experimental conditions within the same parameter space boundary. The simulated random sampling strategy yielded a mean optimal value of 52.95% (standard deviation = 0.63%, 95% confidence interval: 52.91%–52.99%). This means that the XGBoost model cannot match the AL-SSBO prediction values without iterating.

The retrospective assessment based on the cumulative dataset after each AL-SSBO round is presented in Table S4. The mean model-predicted optimum across the bootstrap resamples increased from 49.770 wt% for the initial CCD dataset to 54.013 wt% after Round 5, with the corresponding 95% bootstrap percentile intervals ranging from 48.586 to 50.950 wt% and from 52.764 to 55.264 wt%, respectively. At Round 5, the relative interval width and coefficient of variation of the model-predicted optimum were 4.629% and 1.029%, respectively. Rounds 4 and 5 identified the same best-performing condition: 11.93 h, 68.56 °C, 8.93 wt% lipase loading, and an oil-to-glycerol weight ratio of 11.71. The cumulative best experimental DAG remained at 53.870 wt% from Round 4 to Round 5, giving a Round 5 gain of 0.000 percentage points, below the threshold of 0.604 percentage points defined by the pooled standard error of a triplicate condition mean. The absolute one-step prediction error in Round 5, calculated from the prediction recorded before the round and the corresponding experimental mean, was 0.100 percentage points, below the pooled within-condition standard deviation of 1.046 percentage points. These results met the three retrospective checks and were consistent with stabilization within the predefined search domain under the five-round experimental budget.

### 3.4 RSM and AL-SSBO effect comparison

Table 5 compares the optimal conditions and corresponding experimental validation results obtained using RSM and AL-SSBO. C8 identified the optimal factor combination, and C10 provided the same condition after the model had been updated.

**Table 5:** Comparison of optimal solutions and validation results.

Parameter	RSM (second-order polynomial)	AL-SSBO (XGBoost)
Predicted optimal parameters	Time: 11.78 h Temperature: 70.96 °C Lipase loading: 9.00 wt% Substrate weight ratio: 11.23	Time: 11.93 h Temperature: 68.56 °C Lipase loading: 8.93 wt% Substrate weight ratio: 11.71
Predicted maximum DAG content	53.13	53.87 (C10)
Actual maximum DAG content	51.70	53.77 (C10)
Model error	1.43	0.10

RSM (second-order polynomial) predicted the optimal combination of parameters as follows: time (11.78 h), temperature (70.96 °C), lipase loading (9.00 wt%), and substrate weight ratio (11.23), with a predicted DAG content of 53.13%. The experimental validation DAG content was 51.70 wt%, corresponding to an absolute prediction error of 1.43 percentage points. At this validation condition, the prediction exceeded the experimental result.

Compared to the RSM, the AL-SSBO framework with XGBoost identified the following optimal parameter set: time (11.93 h), temperature (68.56 °C), lipase loading (8.93 wt%), and substrate weight ratio (11.71). The predicted DAG content was 53.87 wt%, compared with an experimental mean of 53.77 wt% from the independent triplicate reevaluation at C10. The absolute prediction error at this condition was 0.10 percentage points.

The sequential and adaptive nature of the AL-SSBO process generates observations that are neither independent nor identically distributed, which violates the i.i.d. assumption underlying standard parametric tests like the *t*-test. To provide a robust demonstration of AL-SSBO over conventional RSM, bootstrap-based inference was employed instead of classical null-hypothesis significance testing. This approach is methodologically sound, as it accounts for the non-stationary structure of the data and thus enables valid and reliable comparative inference. The bootstrap-based inference distribution of the optimal DAG content was estimated using data from the final 3 rounds, yielding a bootstrap mean of 53.47 (standard deviation (SD) = 0.35). The bootstrap-based inference distribution of the AL-SSBO optimum yielded a 95% interval of 52.77–54.16 wt%, which was higher than the experimentally observed RSM optimum of 51.70 wt%. Furthermore, the estimated probability of falling within the region of practical equivalence (ROPE), defined as being within ±0.5% of the RSM, was 0. The standardized effect size (Cohen's d = 2.88) indicates strong statistical confidence and meaningful practical improvement. The evaluation shows that AL-SSBO is superior to RSM for process optimization.

### 3.5 AL-SSBO advantage analysis

This study systematically examined the adaptive optimization mechanisms, parameter learning, and spatial concentration of active learning combined with AL-SSBO. The aim was to clarify the underlying mechanisms by which AL-SSBO enables the efficient identification of optimal reaction conditions.

Fig. 3A illustrates the temporal dynamics of the exploration–exploitation trade-off in AL-SSBO. The algorithm exhibited a peak in the exploration score (distance) during the exploration phase in Round 2, followed by an 80% decline in Round 3. Concurrently, the exploitation score (improvement) increased from 0.021 to 1, indicating a decisive transition. By Round 5, the exploration score (distance) had diminished to 0.064, indicating increased concentration of the recommended conditions near the high-performing region rather than establishing a prespecified convergence endpoint.

**Fig. 3:**
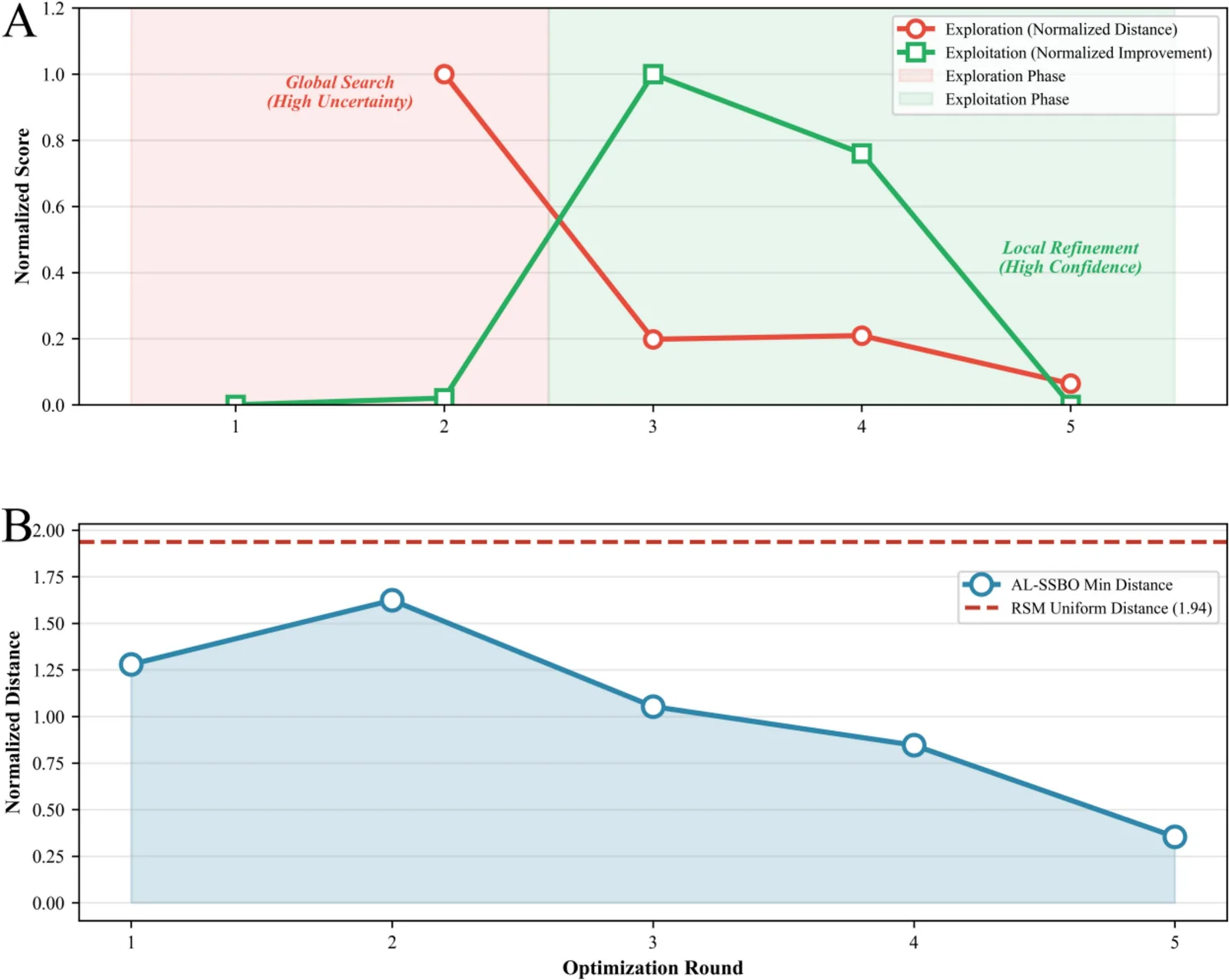
Adaptive optimization mechanisms in AL-SSBO: (A) Exploration-exploitation trade-off dynamics. (B) Adaptive distance strategy.

Combined with the performance of Round 5 Sample 1 (51.60%), these findings demonstrated that AL-SSBO is designed to be cautious: the algorithm recommended a distant verification point (exploration score distance = 0.988) to ensure that no superior region was overlooked, even if this resulted in poorer performance. This contrasts with the static design of RSM, which cannot allocate such experiments adaptively.

Fig. 3B illustrates AL-SSBO's adaptive spatial sampling strategy using normalized distances to known optima. Starting from Round 1 (distance = 1.279, representing 66% of the RSM), the algorithm performed a controlled exploratory expansion in Round 2 (distance = 1.624, representing 84% of the RSM). This was followed by monotonic contraction through Rounds 3–5 (decreasing from 1.053 to 0.354). Notably, the distance in Round 5 represents only 18% of the RSM uniform-design threshold (1.937), demonstrating the transition from global exploration to local exploitation.

To quantify the contribution of the K-Means diversification strategy, the spatial dispersion of the sampled points within the parameter space was analyzed. The AL-SSBO framework achieved an actual spatial dispersion of 2.845. This substantial improvement in spatial coverage empirically validates the critical role of K-means clustering in operationalizing the exploration–exploitation trade-off. By systematically partitioning the design space and enforcing sampling diversity across distinct regions, the algorithm mitigates the risk of overconcentration in local optima while maintaining focus on areas of high potential as identified by the surrogate model. This maximizes the amount of information obtained per experiment and solidifies the robustness of the optimization outcome.

Together, these results indicated a roundwise shift from broad exploration to localized exploitation within the predefined experimental domain. This sampling pattern was consistent with retrospective spatial stabilization, but did not establish a general convergence rate or stopping criterion.

Fig. 4 summarizes the roundwise sampling trajectory and spatial concentration of the AL-SSBO workflow within the investigated four-parameter design space. The parallel-coordinates plot in Fig. 4A shows the relationships between reaction time, temperature, lipase loading, substrate weight ratio, and DAG content across the 10 recommended conditions, each measured in triplicate. Higher DAG contents occurred mainly at lower temperatures and longer reaction times within the investigated domain.

**Fig. 4:**
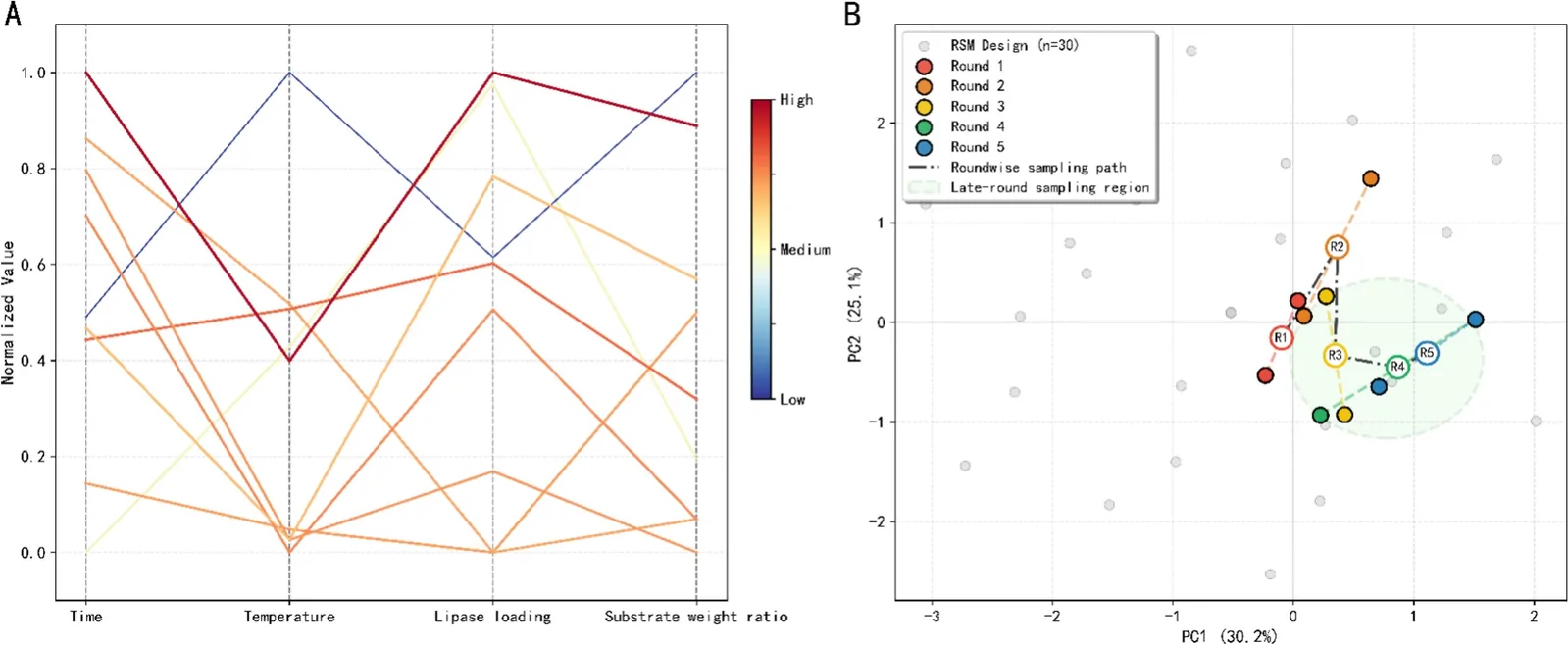
Parameter learning and spatial concentration in AL-SSBO: (A) DAG parallel coordinate plot in AL-SSBO. (B) Spatial concentration trajectory in PCA space.

Fig. 4B and Table 6 show the sampling trajectory of the AL-SSBO algorithm in two-dimensional principal component (PC) space derived from a principal component analysis of the four process parameters. PC1 and PC2 collectively account for 55.4% of the total variance, contributing 30.2% and 25.1%, respectively. Loadings analysis indicates that PC1 is predominantly driven by lipase loading (0.636), substrate weight ratio (0.616), and time (0.449), while exhibiting only a weak negative association with temperature (−0.118). Thus, PC1 can be interpreted as a composite factor reflecting the combined influence of lipase dosage and substrate availability. In contrast, PC2 is dominated almost entirely by temperature (0.950), with a modest positive loading from the substrate weight ratio (0.275) and negligible loadings for time (0.014) and lipase loading (−0.147). This supports the interpretation of PC2 as a temperature-dominated component. The 5 rounds AL-SSBO trajectory delineates a strategic exploration–concentration pattern. Round 1 starts near the origin at (−0.10, −0.16). Round 2 then diverges markedly toward the positive PC2 direction, reaching (0.37, 0.75), a clear exploration of elevated temperature regimes. Round 3 rapidly retraces this step, shifting to (0.35, −0.33) as PC2 turns negative, indicating that the high-temperature region was swiftly deprioritized. Rounds 4 and 5 then progress steadily along the positive PC1 axis, advancing from (0.87, −0.45) to the final-round sampling location at (1.11, −0.31). This late-round sampling region, characterized by high PC1 and low PC2, corresponds to experimental conditions involving elevated lipase loading, a high substrate weight ratio, an extended reaction time, and a reduced temperature. This interpretation is consistently supported by the parallel coordinate plot in Fig. 4A.

**Table 6:** Principal component loadings of process parameters for AL-SSBO.

Principal component	Time(h)	Temperature (°C)	Lipase loading (wt%)	Substrate weight ratio
PC1	0.449	−0.118	0.636	0.616
PC2	0.014	0.950	−0.147	0.275

The observed sampling pattern was consistent with a two-stage search strategy. The early rounds sampled the high-temperature region, whereas the later rounds concentrated on lower-temperature conditions associated with higher experimental DAG contents. Compared with the initial RSM design (σ = 1.250), the final-round AL-SSBO sample set had a lower descriptive standard deviation (σ = 0.543), corresponding to a 56.6% reduction in this dispersion measure. This reduction was consistent with increasing spatial concentration in the later rounds, but was not interpreted as a formal test of convergence.

The observed trends can be explained by the fundamental kinetic mechanisms governing lipase-mediated esterification and glycerolysis. Temperature has a dual influence on DAG yield. Within the low or moderate temperature range, increasing temperature accelerates molecular motion and enhances the frequency of effective collisions between the active sites of lipase and the substrate molecules. This elevates the reaction rate and promotes DAG accumulation. However, lipases are protein biocatalysts that possess thermal sensitivity. Once the temperature surpasses a critical threshold, leading to a sharp decline in DAG content. Reaction time is a critical factor in the attainment of thermodynamic equilibrium. DAG synthesis is a reversible process involving multiple sequential and parallel steps, including the hydrolysis of triglycerides, the esterification of glycerol with free fatty acids, and the migration of acyl groups between positional isomers. Prolonging the reaction time may enable the enzymatic system to approach thermodynamic equilibrium, thereby maximizing the steady-state concentration of DAG before competing side reactions or product degradation become dominant. Therefore, within the investigated reaction time range, extending the reaction time may have favored DAG accumulation by allowing the glycerolysis reaction to proceed further.

Furthermore, the synergistic interplay between temperature, the weight ratio of the substrate, and the lipase is rooted in the kinetics of multi-substrate enzymatic reactions. Increasing the temperature elevates the kinetic energy of all molecules, thereby amplifying the effective utilization of both the catalytic capacity of the lipase and the availability of the substrate. A high substrate weight ratio shifts the reaction equilibrium toward ester synthesis, thermodynamically favouring the formation of DAG. Concurrently, increased lipase loading raises the concentration of available active sites. When these factors work together, maximizing the catalytic efficiency of abundant lipase molecules and promoting the effective collision and conversion of the enriched substrate pool. This synergistic effect results in a significant increase in DAG yield, which exceeds what would be predicted from the additive contributions of the individual factors.

The iterative optimization trajectory of the AL-SSBO model aligns with these well-established principles of enzymatic kinetics, demonstrating its ability to accurately simulate the underlying reaction system and reliably identify process conditions that maximize DAG content. The model captures optimal parameter combinations and reflects mechanistic interdependencies among reaction variables, making it a powerful tool for optimization.

The analysis shows that AL-SSBO gradually identifies the optimal solution through self-learning across multiple iterations. With a small number of samples, it achieves a stable increase in sunflower seed oil DAG content and efficient utilization of resources. Compared to RSM, the AL-SSBO method is more efficient in optimization and resource usage.

### 3.6 Discussion

In this study, using the optimization of DAG synthesis conditions as a case study, we experimentally implemented and evaluated an AL-SSBO workflow and systematically compared it with RSM. As shown in Table 5, AL-SSBO not only identified superior process conditions—which boosted the target yield by 2.07% relative to RSM—but also exhibited lower prediction errors, with RSM's error being 14 times that of AL-SSBO. Bootstrap analysis showed that the interval for the AL-SSBO optimum was higher than the experimentally observed RSM optimum, supporting the observed performance advantage. These findings indicate that, even with the data provided by the CCD, the RSM model, based on a quadratic polynomial assumption, had nearly reached the limit of its predictive capability and struggled to accurately capture the local optimum. In contrast, AL-SSBO, benefiting from a surrogate model and an active learning mechanism, was still able to uncover latent improvement potential.

To rigorously evaluate AL-SSBO, we adopted the fairest possible baseline, thus enabling both AL-SSBO and RSM to commence optimization from exactly the same set of CCD initial samples. However, this strict fairness constraint may actually obscure some substantive advantages of AL-SSBO. Specifically, the CCD sample set already lay within a near-optimal range established through extensive prior single-factor experiments, and with this information-rich starting point, RSM could already approach the upper bound of its fitting and predictive capacity. Therefore, although AL-SSBO improved the yield over RSM by only 2.07 percentage points, this incremental breakthrough was achieved via five optimization rounds comprising 10 recommended evaluations, in a near-optimal region where RSM had nearly exhausted its modeling capability. More importantly, the AL-SSBO framework is not inherently restricted to a CCD-based initial dataset and may be applied using other initial experimental designs. Its performance with sparse, non-orthogonal, or less informative initial datasets should be evaluated in future studies, particularly for optimization systems lacking prior single-factor experiments.

The performance advantage of AL-SSBO also stems from its more comprehensive exploitation of sample information. RSM was fitted to the initial CCD dataset, whereas AL-SSBO incorporated the individual replicate measurements obtained under sequentially recommended conditions into subsequent model updates. Combined with surrogate model, thereby better balancing exploration of uncharted space and fine exploitation of known regions. This not only accounts for the lower prediction error of AL-SSBO evident in Table 5 but also demonstrates its greater iteration rate in Fig. 3.

More importantly, we interpreted the optimization mechanism of AL-SSBO from the perspective of the chemistry. Tracing its 5 rounds sequential iterative trajectory reveals that the new experimental conditions recommended by AL-SSBO were not undirected explorations but progressively concentrated in a region characterized by a lower temperature, a longer reaction time, and higher lipase loading and oil-to-glycerol weight ratio. In other words, the AL-SSBO optimization process is not a purely data-driven black box; its search path aligns closely with the inherent logic of the chemical reaction, thus endowing the optimization with both high efficiency and mechanistic interpretability and greatly enhancing the credibility of the results.

Within the present system, the AL-SSBO workflow provided a flexible few-sample, multi-round optimization strategy that may be extended to multiresponse optimization. However, its transferability and iteration requirements require validation in other experimental systems and with other initial experimental designs. Future studies should therefore assess the number of rounds and total experimental budget required to reach a predefined performance target under different starting datasets. Overall, the present study provides experimental evidence supporting AL-SSBO as an adaptive process-optimization strategy for sunflower oil DAG synthesis.

## 4 Conclusion

This study experimentally implemented a five-round prediction–recommendation–experiment–update workflow for optimizing the enzymatic production of DAG from sunflower oil. GP, RF, and XGBoost were evaluated as surrogate models. RF yielded the lowest shared-dataset MAE, whereas RF and GP shared the lowest shared-dataset RMSE at the reported precision. Across the separately realized optimization trajectories, XGBoost yielded the lowest final-round sequential MAE and RMSE and the highest experimentally observed DAG content. XGBoost was therefore retained for the implemented workflow without claiming universal model superiority. Within the investigated experimental domain, the workflow identified a parameter combination of 11.93 h, 68.56 °C, 8.93 wt% lipase loading, and an oil-to-glycerol weight ratio of 11.71, which produced 53.77 wt% DAG. The final-round results were consistent with stabilization under the predefined five-round experimental budget, although they do not constitute a general convergence criterion.

These conditions provide a laboratory-scale process-development reference for Novozym 435-catalyzed glycerolysis of sunflower oil rather than a directly transferable industrial operating specification. Subsequent work should validate the workflow during process scale-up, determine the effects of repeated lipase use on reaction reproducibility and process cost, and extend the sequential strategy to multiobjective optimization incorporating DAG yield, MAG and FFA formation, DAG-isomer distribution, energy input, and alternative oil feedstocks.

## CRediT authorship contribution statement

**Haoran Liu:** Writing – review & editing, Writing – original draft, Visualization, Validation, Software, Methodology, Investigation, Formal analysis, Data curation, Conceptualization. **Yu Dai:** Funding acquisition, Formal analysis, Data curation. **Hong Liu:** Resources. **Siyao Zhang:** Visualization, Methodology, Formal analysis, Conceptualization. **Huanjun Yu:** Methodology, Formal analysis, Conceptualization. **Qiqing Liao:** Visualization, Methodology, Conceptualization. **Dong Chen:** Validation, Resources, Project administration, Funding acquisition, Conceptualization. **Xiaoming Jiang:** Project administration, Formal analysis. **Dongping He:** Supervision.

## Declaration of Generative AI and AI-assisted technologies in the writing process

During the preparation of this work, the authors used Kimi, DeepSeek, and DeepL to assist with language translation and polishing in order to improve readability. After using these tools, the authors reviewed and edited the content as needed and take full responsibility for the content of the published article.

## Declaration of competing interest

The authors declare that they have no known competing financial interests or personal relationships that could have appeared to influence the work reported in this paper.

## Data Availability

Data will be made available on request.
