# Supplementary material for: Active learning sequential surrogate-based optimization (AL-SSBO) versus response surface methodology in sunflower oil diacylglycerol synthesis: Performance and insight into AL-SSBO
Source: Food Chem X. 2026 Sep 25;39:104516. doi: 10.1016/j.fochx.2026.104516 (PMC13651641; doi:10.1016/j.fochx.2026.104516)
Supplement: Supplementary file 1 [file mmc1.docx]

**Table S1** Design and results of the CCD.

| Numbers | $\text{X}_{\text{1}}$ | $\text{X}_{\text{2}}$ | $\text{X}_{\text{3}}$ | $\text{X}_{\text{4}}$ | DAG content (%) |
| --- | --- | --- | --- | --- | --- |
| 1 | -2 | 0 | 0 | 0 | 47.48 |
| 2 | -1 | -1 | -1 | -1 | 45.12 |
| 3 | -1 | -1 | -1 | 1 | 43.05 |
| 4 | -1 | -1 | 1 | -1 | 43.86 |
| 5 | -1 | -1 | 1 | 1 | 44.97 |
| 6 | -1 | 1 | -1 | -1 | 47.23 |
| 7 | -1 | 1 | -1 | 1 | 47.96 |
| 8 | -1 | 1 | 1 | -1 | 48.84 |
| 9 | -1 | 1 | 1 | 1 | 49.02 |
| 10 | 0 | -2 | 0 | 0 | 43.45 |
| 11 | 0 | 0 | -2 | 0 | 46.44 |
| 12 | 0 | 0 | 0 | -2 | 43.10 |
| 13 | 0 | 0 | 0 | 0 | 52.52 |
| 14 | 0 | 0 | 0 | 0 | 52.25 |
| 15 | 0 | 0 | 0 | 0 | 53.34 |
| 16 | 0 | 0 | 0 | 0 | 51.48 |
| 17 | 0 | 0 | 0 | 0 | 51.66 |
| 18 | 0 | 0 | 0 | 0 | 52.20 |
| 19 | 0 | 0 | 0 | 2 | 43.14 |
| 20 | 0 | 0 | 2 | 0 | 50.91 |
| 21 | 0 | 2 | 0 | 0 | 47.60 |
| 22 | 1 | -1 | -1 | -1 | 44.50 |
| 23 | 1 | -1 | -1 | 1 | 47.20 |
| 24 | 1 | -1 | 1 | -1 | 48.08 |
| 25 | 1 | -1 | 1 | 1 | 51.11 |
| 26 | 1 | 1 | -1 | -1 | 46.63 |
| 27 | 1 | 1 | -1 | 1 | 46.72 |
| 28 | 1 | 1 | 1 | -1 | 48.35 |
| 29 | 1 | 1 | 1 | 1 | 50.35 |
| 30 | 2 | 0 | 0 | 0 | 49.44 |

**Table S2** Optimization results for GP- and RF-based AL-SSBO.

| Model | Round | Point | Time  (h) | Temperature  (°C) | Lipase loading  (%) | Weight ratio | Predicted DAG  (wt%) | Rep 1 | Rep 2 | Rep 3 | Mean | Std | RSD (%) |
| --- | --- | --- | --- | --- | --- | --- | --- | --- | --- | --- | --- | --- | --- |
| GP | 1 | C1 | 11.91 | 69.64 | 8.85 | 11.39 | 52.45 | 51.52 | 53.66 | 53.99 | 53.06 | 1.34 | 2.53 |
|  |  | C2 | 11.17 | 74.45 | 8.18 | 11.18 | 52.40 | 49.37 | 52.49 | 51.61 | 51.16 | 1.61 | 3.14 |
|  | 2 | C3 | 11.20 | 70.95 | 9.00 | 10.9 | 52.23 | 51.50 | 52.44 | 53.11 | 52.35 | 0.81 | 1.54 |
|  |  | C4 | 11.09 | 66.83 | 8.31 | 10.98 | 51.95 | 51.06 | 51.25 | 49.82 | 50.71 | 0.78 | 1.53 |
|  | 3 | C5 | 11.37 | 70.10 | 8.82 | 11.18 | 52.98 | 53.41 | 52.45 | 51.78 | 52.55 | 0.82 | 1.56 |
|  |  | C6 | 11.90 | 68.91 | 8.69 | 11.09 | 52.53 | 53.11 | 53.43 | 52.83 | 53.12 | 0.30 | 0.57 |
|  | 4 | C7 | 11.23 | 70.77 | 8.72 | 11.33 | 52.49 | 53.12 | 50.87 | 53.34 | 52.44 | 1.37 | 2.61 |
|  |  | C8 | 11.37 | 76.68 | 8.94 | 11.01 | 52.25 | 49.81 | 50.38 | 50.88 | 50.36 | 0.54 | 1.06 |
|  | 5 | C9 | 11.62 | 69.38 | 8.2 | 11.13 | 52.55 | 52.48 | 53.43 | 52.99 | 52.97 | 0.48 | 0.90 |
|  |  | C10 | 11.70 | 63.47 | 8.75 | 11.24 | 52.26 | 49.01 | 50.14 | 49.38 | 49.51 | 0.58 | 1.16 |
| RF | 1 | C1 | 11.47 | 70.94 | 7.6 | 10.54 | 52.33 | 49.41 | 50.21 | 50.05 | 49.89 | 0.42 | 0.85 |
|  |  | C2 | 11.11 | 66.28 | 7.52 | 10.95 | 52.33 | 50.37 | 50.68 | 50.23 | 50.43 | 0.23 | 0.46 |
|  | 2 | C3 | 10.98 | 72.27 | 7.79 | 11.1 | 52.36 | 50.42 | 51.48 | 52.55 | 51.48 | 1.07 | 2.07 |
|  |  | C4 | 10.53 | 65.88 | 8.12 | 11.31 | 52.23 | 47.81 | 49.38 | 50.88 | 49.36 | 1.54 | 3.11 |
|  | 3 | C5 | 11.06 | 69.32 | 8.36 | 11.24 | 51.74 | 51.90 | 53.54 | 53.09 | 52.84 | 0.85 | 1.60 |
|  |  | C6 | 11.05 | 75.14 | 8.87 | 10.81 | 51.43 | 52.25 | 49.92 | 51.68 | 51.28 | 1.21 | 2.37 |
|  | 4 | C7 | 11.23 | 70.50 | 8.19 | 10.73 | 52.18 | 52.24 | 52.38 | 52.66 | 52.43 | 0.21 | 0.41 |
|  |  | C8 | 11.16 | 65.52 | 7.94 | 10.58 | 52.03 | 49.85 | 50.33 | 52.62 | 50.93 | 1.48 | 2.91 |
|  | 5 | C9 | 10.76 | 65.25 | 8.24 | 10.78 | 52.14 | 51.26 | 50.48 | 50.21 | 50.65 | 0.55 | 1.08 |
|  |  | C10 | 11.08 | 68.61 | 8.57 | 11.18 | 52.15 | 51.40 | 51.69 | 52.07 | 51.72 | 0.34 | 0.65 |

GP, Gaussian process; RF, random forest. Each model recommended two experimental conditions per round for five rounds, and each condition was evaluated in triplicate. Experimental DAG contents are presented as mean ± SD. Prediction error was calculated as the experimental mean minus the model prediction obtained before experimental evaluation. Absolute prediction error represents the absolute value of this difference. pp, percentage points.

**Table S3** Comparison of prediction errors and iterative optimization performance among XGBoost, GP, and RF surrogate models.

| Model | Iterative round | Shared-dataset CV MAE  (mean ± SD, pp) | Shared-dataset CV RMSE  (mean ± SD, pp) | Sequential MAE  (pp) | Sequential RMSE  (pp) | Cumulative best DAG  (wt%) | Iterative improvement rate (pp/round) |
| --- | --- | --- | --- | --- | --- | --- | --- |
| XGBoost | 1 | — | — | 2.33 | 2.5 | 52.24 | 0 |
|  | 2 | — | — | 3.06 | 4.03 | 52.24 | 0 |
|  | 3 | — | — | 1.03 | 1.07 | 52.76 | 0.52 |
|  | 4 | — | — | 0.46 | 0.6 | 53.87 | 1.11 |
|  | 5 | — | — | 0.17 | 0.18 | 53.87 | 0 |
|  | Overall | 2.02 ± 0.46 | 2.68 ± 0.63 | 1.41 | 2.19 | — | — |
| GP | 1 | — | — | 0.92 | 0.98 | 53.06 | 0.82 |
|  | 2 | — | — | 0.68 | 0.88 | 53.06 | 0 |
|  | 3 | — | — | 0.51 | 0.52 | 53.12 | 0.06 |
|  | 4 | — | — | 0.97 | 1.34 | 53.12 | 0 |
|  | 5 | — | — | 1.58 | 1.96 | 53.12 | 0 |
|  | Overall | 1.64 ± 0.38 | 2.24 ± 0.61 | 0.93 | 1.24 | — | — |
| RF | 1 | — | — | 2.18 | 2.19 | 52.24 | 0 |
|  | 2 | — | — | 1.88 | 2.13 | 52.24 | 0 |
|  | 3 | — | — | 0.63 | 0.79 | 52.84 | 0.6 |
|  | 4 | — | — | 0.67 | 0.79 | 52.84 | 0 |
|  | 5 | — | — | 0.96 | 1.1 | 52.84 | 0 |
|  | Overall | 1.62 ± 0.44 | 2.24 ± 0.61 | 1.26 | 1.53 | — | — |

XGBoost, extreme gradient boosting; GP, Gaussian process; RF, random forest; CV, cross-validation; MAE, mean absolute error; RMSE, root mean square error; pp, percentage points. Shared-dataset CV results are presented as mean ± SD and were calculated using the same dataset and cross-validation partitions for all three models. Sequential MAE and RMSE were calculated from the two conditions recommended by each model in each round; overall values were calculated from all ten recommended conditions. Cumulative best DAG content represents the highest experimental DAG content observed up to that round. Iterative improvement rate was calculated as the increase in cumulative best DAG content relative to the preceding round, expressed in pp/round.

**Table S4** The retrospective assessment based on the cumulative dataset after each AL-SSBO round

| Iterative round | Cumulative observations (n) | Bootstrap optimum (wt%; 95% CI) | Relative CI width (%) | CV (%) | Cumulative best DAG (wt%) | Gain (pp) |
| --- | --- | --- | --- | --- | --- | --- |
| 0 | 30 | 49.770 [48.586, 50.950] | 4.749 | 1.217 | 52.24 | — |
| 1 | 36 | 50.437 [49.336, 51.324] | 3.943 | 1.014 | 52.24 | 0.00 |
| 2 | 42 | 51.543 [50.669, 52.343] | 3.249 | 0.808 | 52.24 | 0.00 |
| 3 | 48 | 51.299 [50.106, 52.193] | 4.068 | 1.026 | 52.76 | 0.52 |
| 4 | 54 | 53.550 [52.065, 54.548] | 4.638 | 1.156 | 53.87 | 1.11 |
| 5 | 60 | 54.013 [52.764, 55.264] | 4.629 | 1.029 | 53.87 | 0.00 |

pp, percentage points. Cumulative best DAG represents the highest round-specific experimental mean obtained up to the indicated round. Gain represents the difference between the cumulative best DAG values of the current and preceding rounds.

**Fig. S1.** Effects of single factors on DAG content: (A) Time. (B) Temperature. (C) Lipase loading. (D) Weight ratio. In each panel, only the indicated factor was varied, while the other three factors were held at their respective center-point levels. The center-point levels were 10 h for time, 70 °C for temperature, 8 wt% for lipase loading, and 11 for the weight ratio. Error bars represent standard deviation (n = 3).

**Fig. S2.** Prediction error and iterative optimization performance of XGBoost, GP, and RF surrogate models: (A) Round-specific mean absolute prediction error. (B) Cumulative best experimental DAG content across optimization rounds.
